# HealthCare Complexity Index (HCIn): A Clinical Decision-Making Tool for Primary Care

**DOI:** 10.3390/healthcare14182976

**Published:** 2026-09-11

**Authors:** Enrique Monsalvo-San Macario, Andrea Sierra-Ortega, Rosa Fernández-Fernández, Verónica Sánchez-Niño, Almudena del Puerto-Claros, Juan Antonio Sarrión-Bravo, Alexandra González-Aguña, Jose María Santamaría-García

**Affiliations:** 1 Department of Computer Science, University of Alcala, 28805 Madrid, Spain; enrique.monsalvo@salud.madrid.org; 2 Hospital Care Research Group (iCuHo), Health Research Institute of La Princesa University Hospital, Madrid Health Service, 28006 Madrid, Spain; rfernandezf@salud.madrid.org (R.F.-F.); vsanchezn@salud.madrid.org (V.S.-N.); almudena.puerto@salud.madrid.org (A.d.P.-C.); jantonio.sarrionb@salud.madrid.org (J.A.S.-B.); alexandra.gonzalez@salud.madrid.org (A.G.-A.); josem.santamaria@salud.madrid.org (J.M.S.-G.); 3 Juan de Austria Health Centre, Community of Madrid Health Service (SERMAS), 28804 Madrid, Spain; 4 Academia Sorge, Travesía de San Roque, 25, 19002 Guadalajara, Spain; 5 Department of Nursing and Physical Therapy, University of Alcala, 28805 Madrid, Spain; 6 Meco Health Centre, Community of Madrid Health Service (SERMAS), 28880 Madrid, Spain; 7 Primary Care Management, Eastern Healthcare Directorate (SERMAS), 28803 Madrid, Spain; 8 Technical Directorate for Project Integration and Control: Primary Care Management Office, Community of Madrid, Spain, 28035 Madrid, Spain; 9 Santa Cristina University Hospital, Community of Madrid Health Service (SERMAS), 28009 Madrid, Spain; 10 Primary Care Management, Multidisciplinary Teaching Unit, Eastern Area, 28017 Madrid, Spain

**Keywords:** primary Health CARE, electronic health records, healthcare innovation, multimorbidity, patient-centered care, risk Index, personalized care

## Abstract

Background: Population ageing, multimorbidity, and chronic conditions have increased the demand for person-centred care in Primary Health Care. Current stratification systems rely on diagnoses or resource utilization and fail to capture the multidimensional nature of care complexity, which also depends on functional, social, environmental factors. Objective: To develop a HealthCare Complexity Index using routinely collected electronic health record (EHR) data and the Person-Centred Care Knowledge Model. Methodology: A retrospective, cross-sectional observational study was conducted in Primary Health Care in the Community of Madrid (Spain). Deductive Care Methodology was used to identify functional assessment variables associated with four care dimensions: vulnerability, risk, etiology, and signs/symptoms. A multidisciplinary expert panel selected and validated the variables through iterative consensus rounds. A weighted scoring algorithm, the Care Complexity Index, was designed to quantify care complexity on a 0–100 scale. Relationships between the selected variables and NANDA-I nursing diagnoses were established and validated by an expert in standardized nursing languages. Results: 27 assessment variables were identified and classified into the 4 dimensions of the care model. These variables were integrated into a weighted complexity index with five severity levels ranging from low to critical complexity. Furthermore, 48 NANDA-I nursing diagnoses were linked to the selected variables. Conclusions: The HealthCare Complexity Index provides a standardized, person-centred approach for estimating care complexity using routinely available (EHR) data. It may support early identification of individuals with complex care needs, facilitate individualized care planning, and strengthen clinical decision-making. Future studies should validate its predictive performance and implementation in other clinical practice.

## 1. Introduction

The progressive aging of the population is one of the main challenges facing contemporary health care systems. Increased life expectancy—a result of advances in public health, prevention, and health care—has been accompanied by a sustained rise in the prevalence of chronic diseases, multimorbidity, frailty, and functional dependence, shaping a population profile characterized by increasingly complex care needs [1,2,3]. The complexity of care stems from the presence of multiple chronic conditions and the need to coordinate different professionals and services to ensure comprehensive care [4]. In this context, care can be understood as a continuous process through which individuals address their needs throughout life, in relation to their vulnerability and environment [5].

In Spain, the primary care situation is also focused on the general aging of the population, as this is leading to an increase in chronic diseases, which in turn are causing a rise in cases of dependency and the demand [6].

This demographic and epidemiological transition has profoundly altered the pattern of health care utilization, shifting the focus from a model oriented toward the treatment of acute conditions to one based on continuous, comprehensive, and person-centered care, especially in the setting of primary care, where longitudinal care and coordination are essential to meeting the needs of patients with chronic diseases and multimorbidity [7,8,9].

The available evidence shows that multimorbidity increases exponentially with age and is associated with greater use of health care resources, poorer quality of life, functional decline, a heavier treatment burden, and increased mortality, which necessitates a rethinking of traditional models of health care [10].

In this context, primary care plays an essential role as the level of care responsible for ensuring continuity of care, coordination across levels, and longitudinal care for people with complex needs [11,12].

The growing complexity of patients seen in primary care has highlighted the limitations of traditional stratification models based exclusively on diagnosis, disease burden, or the risk of health care resource utilization.

In this context, the Ministry of Health has identified as a strategic priority the development of tools that allow for an objective estimation of care needs in order to guide resource planning and ensure equity in care. However, the approaches currently used in the National Health System, such as Adjusted Morbidity Groups (AMGs) or Diagnosis-Related Groups (DRGs-APR) [12], continue to be based primarily on morbidity, clinical severity, or expected resource consumption, without specifically incorporating the complexity inherent in the care process [13,14,15,16]. In this regard point out that, despite growing institutional interest in measuring the complexity and intensity of care, there is still no consensus definition of the concept nor sufficiently developed instruments that allow for a comprehensive assessment of care needs, especially in settings such as primary care [15,16].

In this regard, the concept of vulnerability has taken on particular significance in recent years, as it is understood as the susceptibility of a person or population to experience adverse health outcomes as a result of the dynamic interaction between biological, functional, psychological, social, and environmental factors—beyond the mere presence of disease [17,18]. It is important to note that vulnerability is a broad concept that encompasses a person’s relationship with their environment and with other people; it is not the same as the concept of fragility, which is often used in the field of complexity, since fragility refers exclusively to a decline in an individual’s ability to adapt and focuses solely on functional capacity [19].

From this perspective, vulnerability is a key determinant of care needs, as it influences people’s ability to cope with health and disease processes, maintain their autonomy, and make appropriate use of available resources. Consequently, high levels of vulnerability are associated with an increased risk of functional decline, poorer clinical outcomes, greater use of health care services, and worse health outcomes [18].

In the field of primary care, Fernández-Batalla et al. developed and validated the Care Vulnerability Index (CVI) as a standardized tool for measuring care-related vulnerability [20]. Subsequently, Sierra-Ortega et al. demonstrated that indicators routinely recorded in electronic health records allow for the estimation of community vulnerability, highlighting the potential of clinical information systems to identify individuals with greater needs for follow-up, care planning, and individualized care. These findings reinforce the view of vulnerability as a direct determinant of care complexity and as an essential element for moving toward more proactive, personalized, and data-driven primary care models [20,21]. Vulnerability is, therefore, a direct determinant of the complexity of care and a key factor in identifying individuals who require more intensive monitoring and care planning [21].

At the same time, the digital transformation of healthcare systems has made electronic health records a strategic source of information for research and healthcare planning. Primary care clinical information systems collect longitudinal clinical, functional, pharmacological, and social data on virtually the entire population, generating a volume of data that goes beyond mere healthcare records and enables the development of population- and individual-level health indicators. In this regard, various studies have demonstrated the usefulness of electronic records for characterizing community vulnerability, identifying health inequalities, and supporting healthcare decision-making through standardized indicators derived from routine clinical practice [15,16].

In Spain, Primary Health Care is also facing the challenges associated with population ageing, which is related to an increasing prevalence of chronic diseases, dependency, and growing healthcare needs. In the Community of Madrid, consultations specifically use Gordon’s model to assess functional patterns, identify alterations and plan individualized care. Social and functional needs can additionally be assessed through standardized instruments and, when required, coordinated with other professionals [22,23].

This approach positions the electronic health record as a tool with great potential to transform aggregated healthcare data into key insights for clinical and population-based management.

International strategies agree that the future of primary care lies in integrating digitalization, data analytics, and clinical decision support as fundamental elements for addressing the rise in chronic conditions and ensuring the sustainability of health care systems. The World Health Organization, in its framework for person-centered integrated care, emphasizes the need for early identification of individuals with the greatest care needs in order to tailor interventions to their individual characteristics [24]. At the same time, recommendations recently published highlight the need to redesign primary care using data-driven models capable of proactively identifying high-risk populations and directing the efficient allocation of resources toward those with the greatest care needs [25].

In Spain, this strategic direction is reflected in the National Health System’s Strategy for Addressing Chronic Conditions and, specifically, in the 2026–2028 Strategic Plan of the Community of Madrid’s Primary Care Management Division, which lists person-centered care, digital transformation, clinical decision support, strengthening home care, and reorganizing care for chronic and frail patients using objective tools that identify the complexity of care and tailor the intensity of care to the actual needs of each individual [26].

In this context, assessing the complexity of care is a necessary step beyond simply measuring vulnerability or clinical risk in isolation. Assessing complexity and vulnerability allows for a comprehensive identification of individuals’ clinical, functional, and social needs, but its value depends on its translation into care processes tailored to those needs. In this regard, integrated care and interprofessional practice facilitate coordination among professionals and services, promoting continuity of care and preventing a fragmented response. Furthermore, placing the individual at the center of the process allows for the incorporation of their preferences, abilities, and social context into care planning [27,28].

Complexity encompasses not only a person’s clinical condition but also their functional status, family and social environment, availability of support, self-care capacity, and support networks, constituting a dynamic phenomenon [26,29,30]. Recent evidence in primary care shows that patient complexity is influenced by clinical, psychological, social, and organizational dimensions, including multimorbidity, socioeconomic level, health literacy, housing conditions, and care coordination [29,30]. Furthermore, it has been reported that patients with multiple chronic conditions and high complexity are at greater risk of adverse outcomes and require care plans tailored to their limited self-care abilities and the contextual barriers that affect their health management [30].

Evidence shows that the early identification of individuals with complex needs facilitates care coordination, improves continuity of care, enhances self-care, and contributes to a more efficient use of available resources, especially among populations with multimorbidity, frailty, or dependency [13,31].

To address this need, the HealthCare Complexity Index was developed—a clinical tool designed to objectively estimate the complexity of care using information available in primary care clinical information systems and the person-centered care knowledge model [32]. This model integrates various dimensions related to person-centered care, such as vulnerability [20,21,24,33], etiology [34], symptomatology [32], and risk [32,35], using data from primary care medical records. Furthermore, it serves as a clinical and organizational decision-support tool that promotes a more efficient and equitable allocation of available resources.

From this perspective, using clinical information systems as a source of objective indicators of complexity presents an opportunity to move toward data-driven primary care—one capable of combining clinical expertise with person-centered care models that support decision-making, improve population stratification, and promote personalized care. Consequently, the development and evaluation of tools capable of quantifying the complexity of care using information routinely recorded in electronic health records represent a priority area of research to address the challenges posed by the increase in chronicity, frailty, and vulnerability in today’s societies.

The main objective is to develop a care complexity index for the primary care setting using a person-centered model. The secondary objective of the study is to identify healthcare problems and classify them according to the NANDA-II taxonomy.

## 2. Materials and Methods

This is an observational, retrospective, cross-sectional study that uses the Deductive Care Methodology (DCM) to draw inferences about logical relationships between primary care medical record fields (information recorded within the shared electronic health record (AP-Madrid)) and the Knowledge Model about Person Care using Karnaugh maps [5,34,35,36].

Scope: The study is conducted in the Community of Madrid (Spain), at the primary care level.

Period: The data collected correspond to the period 2026.

Study Sample: The sample consists of all functional assessment fields in primary care clinical records.

**Figure 1:**
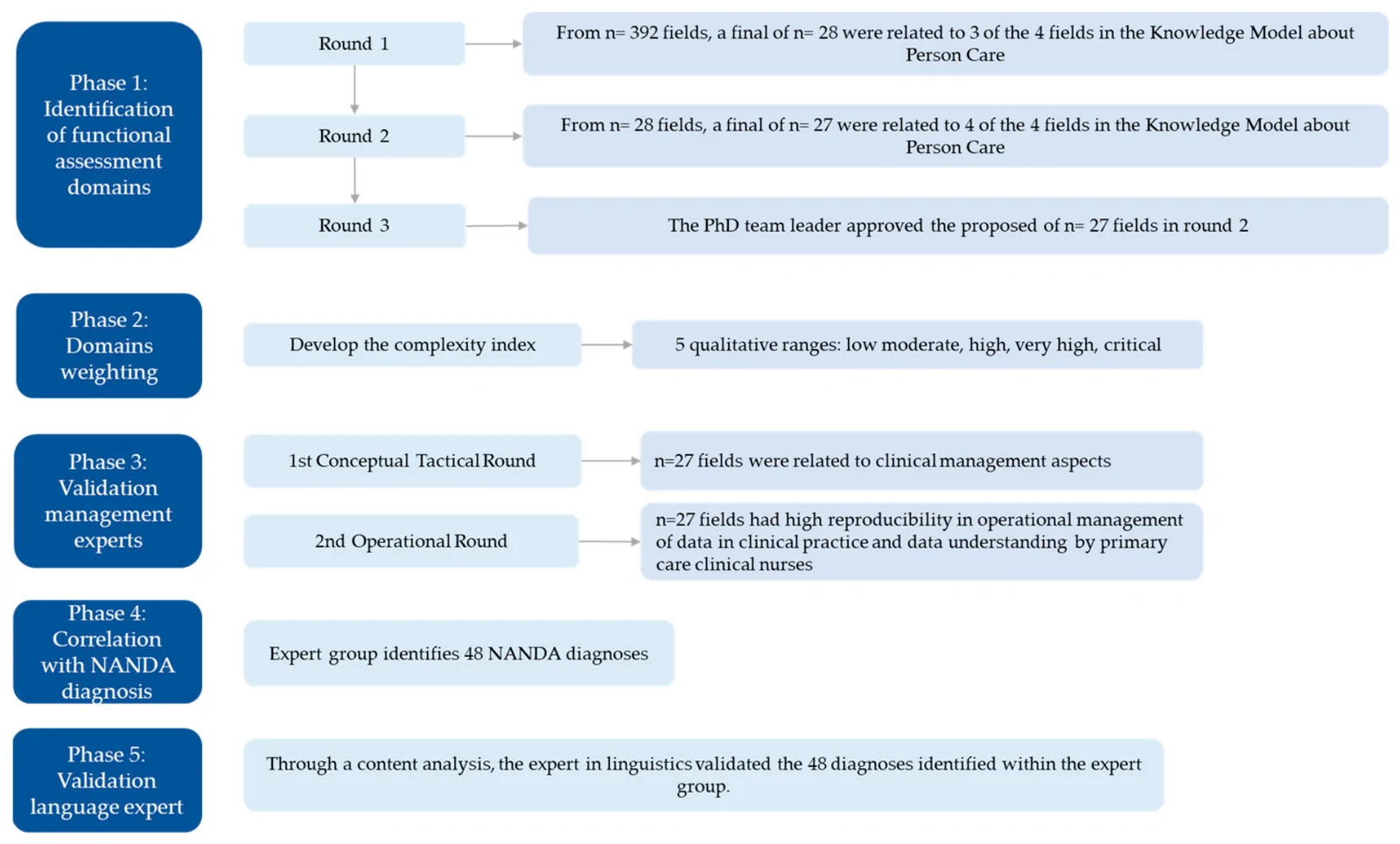
Methodological flowchart.

Process: The research was divided into the following ordered phases: Figure 1 represents the methodological framework used in this study.

### 2.1. Phase 1: Identification of Functional Assessment Domains Related to the Knowledge Model About Person Care

This selection was carried out by a group of experts specializing in family and community care with more than 15 years of experience. The academic background of the group includes a nurse with a Ph.D. in health sciences, a Ph.D. in computer science, and a specialist in family and community; a nurse with a master’s degree in self-care management and a specialist in family and community; and a nurse with a master’s degree in management and administration and specialist in family and community, all of whom are currently providing care in primary care settings.

Using a deductive methodology based on propositional logic via Karnaugh maps [5,34,35,36], only those fields related to the four variables of the Knowledge Model about Person Care [32] were selected from the total of 392 fields in the functional assessment. Specifically, these four variables are:

- Vulnerability: related to the capacity to suffer harm [20,21,32,33].
- Etiology: related to a person’s willingness, knowledge, or ability to perform acts of care [32,34].
- Risk: related to the characteristics of the person’s care environment [5,32,35].
- Symptomatology: related to the presence of acute or chronic health problems in the person [32].

Within the Knowledge Model about Person Care, these fields influence one another, among other factors, and the relationship is as follows:

A person’s vulnerability and risk correspond to their predisposition to develop health problems.

This predisposition, combined with the person’s underlying etiology, gives rise to the person’s potential to develop health problems; if the person also exhibits any symptoms, the potential combined with those symptoms corresponds to the severity of the health problems that a particular person experiences.

Taken together, from the perspective of person-centered care, this is what is known as complexity. Therefore, the sum of each and every field in the model constitutes an assessment of the level of care complexity that a person may experience throughout their life.

Phase 1 was conducted in three rounds by the expert panel.

Round 1: One of the family and community nursing specialists with a master’s degree in management and administration, selected, from a total of 392 fields, a final of 28 that were related to 3 of the 4 fields in the Knowledge Model about Person Care. This resulted in 10 fields related to vulnerability, 0 fields related to etiology, 10 fields related to risk, and 8 fields related to symptomatology.

Round 2: The selection of fields from Round 1 was forwarded for evaluation to a family and community nursing specialist with a master’s degree in self-care management, who reevaluated the results and made changes to the selection. This resulted in a final selection of 27 fields related to the four fields of Knowledge Model about Person Care, specifically 9 fields related to vulnerability, 3 fields related to etiology, 6 fields related to risk, and 9 fields related to symptomatology.

Round 3. The selection made in the second round was presented to the nursing team leader, who holds a Ph.D. in Health Sciences, a Ph.D. in Computer Science, and is a specialist in family and community nursing. The team leader approved the proposed selection, and these fields were established as the final fields.

The final selection of the 27 fields is shown in the results in Table 1.

### 2.2. Phase 2: Weighting the Domains of the Knowledge Model About Person Care

A discrete quantitative value was established to weight each of the four domains of the Knowledge Model about Person Care [32,34,35] in order to estimate the care complexity index.

Predisposition (Pr) 100: Greater weight is assigned to Vulnerability (V; 55) as it represents the person’s baseline condition; Risk (R; 45) modulates vulnerability based on the person’s immediate or distant environment. Thus, Pr = V + R.

Potential (Pt) 100: Greater weight is assigned to Predisposition (Pr; 55), as it is the combination of vulnerability and risk, while the field of Etiology (E; 45) modulates but does not alter the prior baseline, so its assigned weight is lower. Thus, Pt = Pr + E.

Complexity (C) 100: The final set combines Symptomatology (S) with Potentiality (Pt) (taking into account that this includes the previous predisposition values); subsequently, a total weighted score is calculated based on predisposition and potentiality, assigning “double weight” to the base elements at the start of the Logical Cascade, since the significance of the subsequent elements depends on them:

C = V*0.22 + R*0.18 + Pr*0.18 + E*0.14 + Pt*0.12 + S*0.16

(The total of the complexity weighting factors is 100 = 1) Thus, the highest level of complexity would be 100 and the lowest level of complexity would be 0. The mathematical formulation of the formula is available in the Supplementary Materials.

Various HealthCare Complexity Index ranges are established in uniform intervals of 20 points, distributed across the following five qualitative ranges.

HealthCare Complexity Index:

- Low: 0–20;
- Moderate: 21–40;
- High: 41–60;
- Very High: 61–80;
- Critical: 81–100.

### 2.3. Phase 3: Validation of Fields and Care Complexity Index with Management Experts

Two rounds were conducted to reach a final consensus with experts. A tactical round focused on evaluating the management of the complexity tool by the technical management of primary care projects, and an operational round focused on the potential implementation in the primary care clinical setting by the primary care management team in the eastern region.

1st Conceptual Tactical Round: The selection of the 27 fields, derived from applying the care complexity formula, was presented to the technical director of primary care projects. He was given the opportunity to suggest new fields that, based on clinical and management criteria, he believed should be included.

The technical director’s academic background was as follows: registered nurse, Ph.D. in health sciences, specialist in family and community.

During this phase, the technical director of primary care projects verified that the 27 fields were related to clinical management aspects that could be implemented within tactical project management, as they were standard fields in electronic health records, such as the fields for the Norton Scale (related to the risk of developing pressure ulcers) or the Barthel Scale (related to the ability to perform basic activities of daily living), which aligned with the strategic direction of the person centred care outlined in the 2026–2028 Strategic Plan for Primary Care Management [26].

Likewise, it was confirmed that the formula reliably identified the complexity score while taking into account aspects of person-centered care.

Second operational round, in this round, the selection of the 27 fields—based on the application of the care complexity formula—was presented to the management team of the Eastern Primary Care Area of the Community of Madrid, Spain. They were given the opportunity to include new fields that had been excluded during the methodological process but which, based on clinical and management criteria, they considered should be included.

The management consultation team (primary care by nurses specialist) consisted of the director of primary care nursing for the Eastern Area—a specialist in family and community nursing with a master’s degree in management and administration—the nurse in charge of community health for the Eastern Area—a specialist in family and community nursing—and the nurse in charge of the Residential Care Unit—a specialist in family and community nursing.

Final consensus was sought through the “think-aloud” methodology with the management group [37]. The group meeting was structured as follows: PowerPoint presentations by the leaders of the various work streams; a question-and-answer session to ensure that members of the clinical management team understood the material presented.

When the results were presented to the group, they agreed on the selection of 27 fields, noting the high reproducibility both in the operational management of data in clinical practice and in the understanding of that data by primary care clinical nurses. They also found the formula for determining the complexity index to be clear and concise, with high external validity, as it provides several levels of complexity on which clinical staff can focus when providing patient care.

### 2.4. Phase 4: Correlation of the 27 Fields with NANDA Diagnoses

Royal Decree 1093/2010, dated September 3, which approves the minimum data set for clinical reports in the Spain National Health System, states that all data sets in the clinical record’s care report must include a record of nursing diagnoses in NANDA format [38].

Therefore, to identify the care issues represented by the variables of the care complexity index, a mapping was established between the 277 NANDA 2026 diagnoses [39] and the 27 fields.

For convenience, a maximum of two potential NANDA diagnoses was assigned to each of the 27 selected functional assessment fields.

The expert group conducted a content analysis using a propositional logic methodology based on Karnough tables [5,34,35,36], to determine whether the 27 functional assessment domains, within their measurement range (worst-case scenario), were related to any of the 277 NANDA diagnoses.

For example, the domain “level of care,” at its worst-case scenario (“inadequate”), referred to the diagnosis “Ineffective self-management of health”; the domain “home hygiene/safety,” at its worst-case scenario (“inadequate”), referred to the diagnosis “Ineffective home maintenance behaviors.”

A total of 48 NANDA diagnoses were identified that were related to one of the 27 functional assessment fields.

### 2.5. Phase 5: Validation of Diagnoses by a Language Expert

In this phase, the 48 identified NANDA diagnoses were validated by a nursing expert in linguistics. The expert holds a Ph.D. in computer science and a master’s degree in nursing informatics and serves as the head of research at a hospital in the Community of Madrid.

Using content analysis methodology, following a propositional logic approach, it was verified that the fields were related to the proposed diagnoses because they met one of the following criteria: a relationship between the field and the words in the label, or a relationship between the field and the content of the diagnosis definition, for example, for the field “level of care,” at its worst level (“inadequate”), it referred to the diagnosis “Ineffective self-management of health,” because its definition states “unsatisfactory management of symptoms, treatment regimen, and lifestyle changes associated with living with a chronic illness,” in this case, the unsatisfactory management refers to the inadequate level of care. Another example is the “housing hygiene/safety” field, which, at its worst level (“inadequate”), referred to the diagnosis “Ineffective home maintenance behaviors,” because the definition of its label states “Unsatisfactory pattern of knowledge and activities for the safe maintenance of one’s own residence.”

The language expert reviewed the relationships outlined for the 48 selected diagnoses and agreed with them after conducting the content analysis described above.

Of the total of 277 NANDA diagnoses, the 48 diagnoses presented by the expert group and subsequently reviewed by the language expert were ultimately selected.

The table of final diagnoses related to the fields is shown in the Results section in Table 2.

Ethical considerations: No patient data is used, nor are any questionnaires or surveys administered to patients; only the structure of an electronic health record, from which no data is extracted at any point, and, therefore, informed consent is not required.

The research does not collect or identify individual persons (it does not query the Regional Personal Identification Code); at no point during the query performed in the clinical information system regarding the electronic medical record are patient clinical data retrieved. The researchers cannot identify personal data because only the fields comprising the medical records were evaluated, without access to representative data at either the quantitative or qualitative level.

## 3. Results

Each result is related and numbered according to the phases outlined in the methodology.

### 3.1. Results Phase 1: Identification of Functional Assessment Domains Related to the Knowledge Model About Person Care

Table 1 shows the relationship between the 27 fields and the 4 variables of the person-centered care knowledge model. Specifically, 9 fields related to vulnerability, 3 fields related to etiology, 6 fields related to risk, and 9 fields related to symptomatology.

### 3.2. Results of Phase 5: Validation of Diagnoses by Language Experts

Table 2 below shows the relationship between NANDA diagnoses and the 27 fields selected and validated by experts. This resulted in a total of 48 related NANDA diagnoses.

## 4. Discussion

The study demonstrates that care complexity can be estimated on an individualized basis using fields from the functional assessment in primary care medical records. This is key because, in primary care, medical records are standardized and do not depend on the location of care.

Care complexity has been addressed from different perspectives and through instruments incorporating clinical, psychosocial, functional, and organizational dimensions. The INTERMED instrument, for example, conceptualizes complexity from a biopsychosocial perspective and integrates information related to biological, psychological, social, and healthcare-system domains to identify complex healthcare needs [40]. Similarly, Thomas et al. (2016) developed a specific instrument for community nursing that incorporates different dimensions of patients’ situations and care delivery [41]. Perone et al. (2022) also highlighted the need to identify complex patients to facilitate coordinated interprofessional care [42]. In home care, Busnel et al. (2018) likewise conceptualized complexity as a multidimensional phenomenon that extends beyond the clinical situation to include factors related to the person, their environment, and the organization of care [43].

In comparison with these approaches, the present study proposes a different perspective by using exclusively the structured information available in the electronic health record to construct a care complexity index. This approach allows the presence or absence of specific elements recorded in the health record to be identified without requiring the processing of specific numerical values or the administration of additional assessment instruments. Furthermore, unlike approaches focused on professional workload or patient perceptions, the proposed index aims to characterize care complexity based on clinical and health and social care information already available within the healthcare system. This characteristic may facilitate its integration into routine clinical processes and its potential transferability to different healthcare settings with structured electronic health records.

This perspective supports the need to rethink traditional models of healthcare organization, particularly those based on single-disease approaches. Grembowski et al. (2014) [44] conceptualized care complexity as the gap between patients’ needs and the capacity of healthcare services to respond to them, emphasizing that complexity emerges from the interaction between individual, social, and healthcare-system factors. From this perspective, the identification of care complexity should not only describe patients’ needs but also support healthcare systems in recognizing when existing services may be insufficient or poorly aligned with those needs [44]. Our approach contributes to this perspective by using information already available in the electronic health record to systematically identify characteristics associated with greater care complexity, potentially supporting earlier recognition of patients who may require more coordinated and integrated responses.

This approach is not intended to replace individual clinical assessment or to capture all possible dimensions of complexity, but rather to provide a systematic mechanism for identifying complexity using information already generated as part of routine care. In this regard, the use of structured electronic health record fields may support a more reproducible and scalable identification of care complexity, reducing the need for additional assessments and potentially allowing the approach to be adapted to different health information systems. Nevertheless, further studies are required to evaluate its clinical utility and to examine its association with health outcomes, healthcare utilization, care needs, and outcomes reported by patients and professionals. External validation in different healthcare settings will also be necessary to determine the transferability of the proposed approach.

Regarding the utility and generalizability of the study to other settings, our care complexity calculation tool can enhance services for vulnerable populations, such as care for residents in long-term care facilities, or for high-risk care situations requiring an assessment of vaccination status and effective decision-making based on the patient’s care situation. All of this enables individualized care planning and the monitoring of frail chronic patients in both outpatient and home care settings. Furthermore, the developed care complexity tool could potentially support care prioritization and the management of material and human resources, although these potential applications require further clinical validation and evaluation in real-world settings.

Regarding the limitations of the study, the developed HealthCare Complexity Index (HCIn) may contribute to the prioritization of care and to the management of material and human resources. Its use is linked to the development of subsequent validation phases to assess its practical applicability in specific clinical settings, as well as the usability of the computational tool in which the HCIn equation has been implemented.

The HCIn establishes its logical foundation retrospectively and cross-sectionally, as its outcome is determined from causal relationships based on clinical data available at the present time. Its potential predictive applicability should be further investigated, grounding this potential in logical reasoning derived from existing evidence, expert elicitation, or the development of non-retrospective longitudinal studies. This opens up several avenues for future research aimed at strengthening its practical applicability in care planning.

The evidence supporting the HCIn derives from the logic employed, which ensures its theoretical verification. Nevertheless, since it is a novel experimental computational clinical tool, external and prospective validation of its use in real-world settings will be necessary. This validation should involve cases from different healthcare environments in order to assess its generalizability and transferability to other populations, care contexts (such as highly complex populations, home care, or pediatric populations), and healthcare systems, as well as its potential use in the clinical training of healthcare professionals and experts.

Since the selection and weighting of the variables were based on expert judgment and on the theoretical evidence available and considered by the experts, there may be a certain degree of subjectivity in the factual elements incorporated into the HCIn. This may require adapting the index to ensure its generalizability to other clinical settings and healthcare systems. Nevertheless, the logical and algebraic formalization of the HCIn allows for such adaptations without affecting its mathematical architecture.

The HCIn outcome in real-world settings depends on data collected in a standardized manner within electronic health records. Consequently, its performance will depend on the completeness, quality, and standardization of these data in each healthcare setting in which it is implemented.

Another potential avenue for future improvement of its analytical power would be to conduct comparative studies, where applicable, with other instruments for weighting healthcare-associated clinical complexity developed using similar methodologies.

Patient Classification Systems (PCSs) have traditionally been used to classify patients according to their nursing care needs, patient acuity, and associated workload, particularly in hospital settings [45]. Recent studies continue to demonstrate their usefulness for estimating nursing workload and supporting staffing decisions; however, substantial variability exists among systems in terms of their structure, scope, and measurement approaches. For example, Kim et al. (2022) reported that an electronic health record-based Patient Classification System could provide useful information on patient severity, nursing activities, and workload estimation, although some discrepancies were observed when compared with established classification systems [46]. Similarly, a comparison of two Patient Classification Systems showed significant differences in the estimated nursing workload and required staffing for the same patient population, highlighting the influence of the classification approach on the resulting estimates [47]. In addition, Junttila et al. (2023) demonstrated that maintaining the reliability and validity of a PCS in routine clinical practice requires continuous training, auditing, and periodic reassessment, indicating that the validity of these systems cannot necessarily be assumed solely from their implementation [48]. Recent literature has also highlighted limitations in the scope of PCS, particularly their emphasis on measurable nursing workload, with some aspects of nursing care remaining insufficiently captured by conventional classification approaches [49].

Furthermore, a recent scoping review found that evidence directly linking Nursing Patient Classification Systems with patient and care-related outcomes remains limited, with only four studies meeting the review criteria and considerable heterogeneity in the outcomes assessed [50]. Taken together, these findings suggest that, although PCS remain valuable for assessing nursing needs and workload, differences in conceptualization, measurement properties, and their relationship with clinical outcomes support the need for complementary approaches capable of capturing healthcare complexity more comprehensively.

In recent years, there has been a paradigm shift from measuring workload exclusively to assessing the complexity of care. This approach aims to incorporate not only the time spent on interventions but also clinical judgment, decision-making, and the interdisciplinary coordination inherent in the care process.

In other settings, such as hospitals, particularly in highly complex areas like intensive care units, studies have compared the development of tools designed to assess the complexity of care. According to the systematic review by Reguera-Carrasco et al., based on the COSMIN standards, ten different instruments were analyzed, and the authors concluded that the Nursing Activities Score (NAS) was a reliable tool for estimating care complexity through workload [51]. However, this scale places greater emphasis on the activities that the healthcare team must perform rather than on the care characteristics of the individual, so it is not a tool that allows for individualized care planning.

Outside the intensive care setting, the available evidence is more limited. In home care, the review by Flo et al. identified only four classification systems with adequate validity and reliability studies, concluding that there is a significant lack of sufficiently evaluated tools to support resource planning in this care setting [52].

A similar situation is observed in the field of pediatric care. A recent narrative review highlighted the absence of validated instruments specifically designed to measure the complexity of pediatric care, attributing this limitation to a lack of consensus on the very definition of the concept [52]. This conceptual heterogeneity has also been described in the literature on children with complex care needs, where considerable variability in terminology persists [53]. Similarly, recent studies on children with complex care needs highlight that the absence of standardized criteria has limited the consistent identification of this population; furthermore, the characteristics of the child’s primary caregivers must be incorporated when assessing this population—another aspect that complicates reaching consensus on a uniform definition and the development of assessment tools [54].

Overall, the scientific evidence indicates that assessing the complexity of care is a key element in tailoring healthcare organization to patients’ actual needs. In this regard, the literature advocates for the development and implementation of valid, standardized tools that enable the assessment of care complexity across different levels of care, thereby promoting a more precise allocation of resources and care planning centered on each patient’s needs. This need has been described in hospital settings as well as in home care, intensive care units, and pediatric care, where the heterogeneity of care needs highlights the importance of having specific, validated instruments [52,55,56].

## 5. Conclusions

This study presents a HealthCare Complexity Index based on the Person-Centered Knowledge Model and structured data from Electronic Health Records (EHRs). The proposed approach provides a multidimensional and weighted representation of care complexity based on information routinely available in Primary Health Care records.

The logical structure of the HealthCare Complexity Index allows the information contributing to the final score to be decomposed into specific dimensions, including Vulnerability, Risk, Predisposition, Etiology, Potentiality, and Severity. This structure may provide an explanatory perspective on care complexity beyond approaches based solely on diagnostic groupings or healthcare resource utilization.

Furthermore, the use of standardized EHR data may facilitate the linkage of the Index with healthcare taxonomies and Knowledge-Based Systems using standardized terminology. From a computational perspective, the proposed structure could potentially contribute to the development of interoperable Clinical Digital Twins across different healthcare settings and disciplines. However, these potential applications have not been evaluated in the present study.

Similarly, the index may have potential applications in patient classification and in supporting healthcare resource planning according to levels of care complexity. Nevertheless, such applications require further research and clinical validation before their effectiveness or practical utility can be established.

Overall, the HealthCare Complexity Index represents a preliminary approach to the systematic identification of care complexity using structured information from routine clinical records. Further studies are required to assess its external and prospective validity, predictive performance, and applicability across different healthcare settings and populations.

## Figures and Tables

**Table 1:** Identification of functional assessment domains related to the Knowledge Model about Person Care.

Variables	Variables of The Knowledge Model About Person Care
Level of care	Vulnerability
Norton scale	Vulnerability
Motor impairments	Vulnerability
Paralysis, paresis	Vulnerability
Barthel Index	Vulnerability
Cognitive impairments	Vulnerability
Language difficulties	Vulnerability
Perceptual impairments	Vulnerability
Dependence on another person	Vulnerability
Hygiene and safety in the home	Risk
Accidents	Risk
Lack of resources	Risk
Environmental barriers	Risk
Unfavourable family environment	Risk
Healthy behaviours: interest	Etiology
Knowledge	Etiology
Attitude	Etiology
Inability to carry out normal activities	Etiology
Hospital admission	Signs and symptoms
Nasogastric tube	Signs and symptoms
Nutritional status	Signs and symptoms
Impaired integrity	Signs and symptoms
Ostomy	Signs and symptoms
Urinary catheter	Signs and symptoms
Drains	Signs and symptoms
Level of consciousness	Signs and symptoms
Behavioural disturbance	Signs and symptoms

**Table 2:** Relationship between NANDA diagnostic and 27 variables.

27 Variables	1º Diagnostic option NANDA	2º Diagnostic Option NANDA
Level of care	Ineffective self-management of health	Behaviours that are ineffective for maintaining good health
Norton scale	Risk of physical injury	Syndrome characterised by a decline in the ability to look after oneself
Motor impairments	Impaired physical mobility	None
Paralysis, paresis	Risk of ineffective self-management of health	None
Barthel Index	Syndrome characterised by a decline in the ability to look after oneself	Risk of ineffective self-management of health
Cognitive impairments	Impairments in thought processes	Chronic confusion
Language difficulties	Impaired verbal communication	None
Perceptual impairments	Risk of ineffective health-maintenance behaviours	Risk of ineffective thermoregulation
Dependence on another person	Syndrome characterised by a decline in the ability to look after oneself	Impaired physical mobility
Hygiene and safety in the home	Ineffective household maintenance practices	Behaviours that are ineffective for maintaining good health
Accidents	Risk of behaviours that are ineffective for maintaining good health	None
Lack of resources	Risk of ineffective self-management of health	Risk of a decline in participation in leisure activities
Environmental barriers	Risk of ineffective self-management of health	Impaired social interaction
Unfavourable family environment	Dysfunctional family dynamics	Altered patterns of family interaction
Healthy behaviours: interest	Behaviours that are ineffective for maintaining good health	None
Knowledge	Inadequate knowledge about health	Inadequate health literacy
Attitude	Ineffective self-management of health	Behaviours that are ineffective for maintaining good health
Inability to carry out normal activities	Impaired physical mobility	Risk of impaired cardiovascular function
Hospital admission	Risk of physical injury	None
Nasogastric tube	Difficulty swallowing	Risk of ineffective self-management of dry mouth
Nutritional status	Ineffective self-management of excess weight	Ineffective self-management of low weight
Impaired integrity	Impaired tissue integrity	Impaired skin integrity
Ostomy	Risk of impaired gastrointestinal motility	Risk of impaired bowel function
Urinary catheter	Impaired urinary function	Risk of infection
Drains	Excessive fluid volume	Impaired tissue integrity
Level of consciousness	Impaired verbal communication	Impaired thought processes
Behavioural disturbance	Ineffective impulse control	Impaired thought processes

## Data Availability

The data presented in this study are available on request from the corresponding author.

## Supplementary Materials

The following supporting information can be downloaded at: https://www.mdpi.com/article/10.3390/healthcare14182976/s1.
