# Supplementary material for: HealthCare Complexity Index (HCIn): A Clinical Decision-Making Tool for Primary Care
Source: Healthcare (Basel). 2026 Sep 11;14(18):2976. doi: 10.3390/healthcare14182976 (PMC13606558; doi:10.3390/healthcare14182976)
Supplement: Supplementary file 1 [file healthcare-14-02976-s001.zip › healthcare-4475816-supplementary.pdf]

Mathematical formalization of The equation of Complexity (C)

The equation of Complexity (C) presented in the article corresponds to a theoretical model, the Knowledge Model about Person Care, which manages the clinical information of a specific case [5,34-36].

Formally, it is assumed that the entire set of information managed for a specific case, potentially existing, constitutes the whole of a finite, non-limited universe of data.

Under this model, information is obtained through operations involving data or groups of data that are considered Elements.

An Element may be a Unitary Data Item (UD) or a Data Set (DS).

There are four types of DS:

- Primary DS (DSp), consisting of "n" Unitary Data Items (UD), where n is the number of unitary data items.
- Secondary DS (DSs), consisting of "n" DSp, where $n > 1$.
- Tertiary DS (DSt), consisting of "n" DSs, where $n > 1$, or of n DSp and n DSs, where $n \neq 0$.
- Quaternary DS (DSc), consisting of "n" DSp, n DSs, or n DSt, where $n \neq 0$, and one UD.

An element can operate as a Logic Gate (LG) if its presence must necessarily occur (i.e., it must be non-false in the logical environment or different from 0 in the numerical environment), or if it must fall within a specific range. This element has a logical value greater than that of the value of its set.

The equation is solved, and therefore interpreted mathematically, from left to right and from the inside outward with respect to the parentheses.

This order determines the construction of the equation with respect to the logical model of the Knowledge Model about Person Care, where:

$(bcva,...bcv\Omega) \rightarrow V$; $(RFa,...,RF\Omega) \rightarrow R$; $(V \wedge R) \rightarrow Pr$; $(K,SK,W) \rightarrow E$; $(Pr \wedge E) \rightarrow Pt$; $(sa,\dots,s\Omega) \rightarrow S$

if $E \neq false$

and where: $V \wedge R \wedge Pr \wedge E \wedge Pt \wedge S \rightarrow C$

As a clarification, it should be noted that:

Vulnerability (V) is the quality of being susceptible to harm in a person and is determined from a set of Basic Care Variables (BCV); it is a Primary Data Set (DSp).

Risk (R) affects the way a person's health care, needs, and competencies are met, and is estimated from a set of Risk Factors (RF); it is a Primary Data Set (DSp).

Predisposition (Pr) is the quality of being likely to develop a health alteration and is the set formed by V and R; it is a Secondary Data Set (DSs).

Etiology (E) is the trigger of a health problem and is determined from a set of limitations in knowledge (K), skill (SK), or will (W); it is a Primary Data Set (DSp).

Potentiality (Pt) is the quality of being likely to manifest a health alteration and is the set formed by Pr and E; it is a Secondary Data Set (DSs).

Symptomatology (S) is the expression of a health alteration and is determined from a set of signs or symptoms (s). It can only occur if (E) is true; it is a Primary Data Set (DSp).

When performing the algebraic operations, in order to numerically weight the relationship between elements, we start from the premise, as previously mentioned, that the universe of information of C is finite and can therefore, and must, be limited to 1.

Thus, each element must be equal to 1, and all elements taken together must likewise equal 1.

For Primary Data Sets (DSp), the calculation is performed as follows:

$$V=\sum_{i=1}^{n} bcv;\ R=\sum_{i=1}^{n} RF;\ E = \sum_{i=1}^{n} e\ ;\ S = \sum_{i=1}^{n} s$$

For Secondary Data Sets (DSs), the calculation is performed by assigning a slightly greater weight to the element located on the left side of the equation, normalized to 1:

$Pr = ((V \times 0.55) + (R \times 0.45))$;

$Pt = ((Pr \times 0.55) + (E \times 0.45))$

The general equation for C used is:

$$(V \times 0.22) + (R \times 0.18) + (Pr \times 0.18) + (E \times 0.14) + (Pt \times 0.12) + (S \times 0.16) = C$$

It is normalized to 1 through the weighting of 0.22, 0.18, 0.18, 0.14, 0.12, and 0.16 assigned to each of its elements, applying the following premises, as justified above

The elements on the left-hand side of the equation have a greater weighting than those located on the right-hand side.

The greater weighting must take into account the logical level of an element:

CDp > CDs > CDt > CDc: 0.22 > 0.18; 0.18 > 0.14; 0.18 > 0.14.

The decrease in weighting between elements throughout the equation must remain stable: 0.04 units. If a CD has a Logic Gate as one of its Elements, a greater reduction in its weighting value occurs:

Pr = 0.18; Pt = 0.12 (0.18 − (0.04 + 0.02)).

CDp must have a weighting greater than or equal to that of the CDs comprising them:

0.22 and 0.18 ≥ 0.18;

0.18 and 0.14 ≥ 0.12.

The weighting value of a CDs cannot be greater than the lowest weighting value of its constituent CDp:

0.18, the lower weighting value in Pr (0.22, 0.18) = 0.18, the weighting value of Pr;

0.14, the lower weighting value in Pt (0.18, 0.14) = 0.14, the weighting value of Pt.

The weighting value of a CD that incorporates a Logic Gate (LG), a necessary condition for the CD to acquire a value, cannot be greater than the weighting value of the element acting as the Logic Gate:

E is the LG of Pt; therefore, the weighting value of Pt is lower than that of E:

0.14 > 0.12.

The final logical elements account for the remaining weighting resulting from the normalization of the total equation. In C, the final logical element is S, symptomatology; its value depends on the importance of all the preceding data (as occurs in clinical history-taking):

$S = 0.16$; $S = 1 - (0.22 + 0.18 + 0.18 + 0.14 + 0.12)$.

Under this set of conditions, derived from the importance of the Knowledge Model about Person Care, logic, and algebraic operability, the weighting of the elements in the C equation operates in a balanced manner, such that if one weighting value is modified, it proportionally affects the remainder while maintaining the structure of the equation and, consequently, the management of the clinical information of a case.

Once the mathematical architecture of the C equation has been explained, the ranges associated with the value obtained by C are established in quintiles, whose numerical values—0–20, 21–40, 41–60, 61–80, and 81–100—are related bijectively to a defined clinical significance of C through the application of the logical biconditional operator:

0–20 ↔ low C

21–40 ↔ moderate C

41–60 ↔ high C

61–80 ↔ very high C

81–100 ↔ critical C

Developed by the coauthor Dr Santamaria Garcia JM,

Affilation:ICuHo Hospital Care Research Group, Health Research Institute of La Princesa University Hospital, Madrid Health Service, Santa Cristina University Hospital, 28809 Madrid, Spain;
Academia Sorge, Travesia de San Roque, 25, 19002 Guadalajara, Spain Meco Health Centre, Community of Madrid Health Service (SERMAS), 28880 Madrid, SpainPrimary Care Management, Multidisciplinary Teaching Unit, Eastern Area, 28017 Madrid, Spain
